# Ankaferd blood stopper: A novel additional strategy for less experienced gastroenterologists in gastrointestinal bleeding treatment

**DOI:** 10.1097/MD.0000000000038319

**Published:** 2024-05-31

**Authors:** Berk Baş, Ömer Küçükdemirci, Müge Ustaoglu

**Affiliations:** a Department of Gastroenterology, Ondokuz Mayis University, School of Medicine, Samsun, Turkey; b Department of Gastroenterology, Ondokuz Mayis University, School of Medicine, Samsun, Turkey; c Department of Gastroenterology, Ondokuz Mayis University, School of Medicine, Samsun, Turkey.

**Keywords:** Ankaferd, Ankaferd Blood Stopper, endoscopy, hemostasis, non-variceal upper gastrointestinal bleeding, NVUGIB

## Abstract

The Ankaferd Blood Stopper (ABS) proves effective in managing various bleedings, particularly in surgical and dental procedures. This study assesses ABS efficacy endoscopically by less-experienced endoscopists for non-variceal upper gastrointestinal bleeding (NVUGB). Between 2016 and 2021, our hospital’s Gastroenterology Department Endoscopy Unit conducted a retrospective data analysis of 653 patients who underwent endoscopy for NVUGB. The study included 202 patients who underwent endoscopic interventions performed by endoscopists with less than 3 years of experience. Based on the method used for endoscopic hemostasis, we classified those treated with ABS (either alone or as a second method) as group 1. In contrast, we classified patients treated with non-ABS hemostatic methods into Group 2. The study included 202 patients, with 96 (47.52%) in Group 1 and 106 (52.48%) in Group 2. All patients in Group 1 achieved bleeding control, while 4 patients in Group 2 initially did not achieve bleeding control; however, bleeding control was subsequently established following ABS administration. After 1 month of follow-up, mortality occurred in 3 out of 202 patients (1.48%), and all these cases were in Group 2. There is a significant difference in the need for blood transfusion between the groups (*P* < .001). Regarding the bleeding source, bulbus ulcer and gastric cancer were more prevalent in Group 2. On the other hand, although statistical significance was not reached in the comparison of rebleeding rates between groups, numerically, a higher incidence of recurrent bleeding was observed in Group 2 (Group 1: 3 [3.1%], Group 2: 8 [7.5%]; *P* = .167). Additionally, a similar relationship was noted among intensive care admissions (Group 1: 5 [5.2%]; Group 2: 7 [6.6%]; *P* = .675). In the group that used ABS, there were significantly higher rates of hypotension, tachycardia, syncope, and the need for transfusion than in the other group. In medical practice, this distinction often stems from the shared preference of clinicians to use ABS as a salvage method in cases of more severe bleeding. Considering all the findings, it is evident that using ABS through endoscopy in cases of NVUGIB significantly improves procedural success, irrespective of the endoscopist’s experience level.

## 1. Introduction

Upper gastrointestinal tract hemorrhage remains a notable source of mortality and morbidity despite the progress made in endoscopic and medical interventions.^[1,2]^ In studies conducted, researchers have determined that the incidence of hospitalization due to upper gastrointestinal tract bleeding ranges from 40 to 150 per 100,000 individuals.^[3,4]^ The most common causes are peptic ulcer disease, Mallory-Weiss syndrome, erosive gastritis, duodenitis, esophagitis, malignancy, angiodysplasias, and Dieulafoy’s lesions. Managing upper gastrointestinal bleeding can be challenging, depending on the cause and location of the hemorrhage. In managing non-variceal upper gastrointestinal bleeding (NVUGIB), the European Society of Gastroenterology guidelines recommend combining of the 2 methods to achieve hemostasis.^[5]^ The success rate of interventions involving combination methods, such as adrenaline injection followed by a second hemostasis modality, has been found to range from 85% to 95%.^[4,5]^ Hemoclips, argon plasma coagulation (APC), sclerotherapy, heater probe, and hemospray were applied under endoscopic guidance, either individually or in combination. Each of these methods has its advantages and disadvantages, and therefore, the choice of method should be tailored to the individual patient.^[6,7]^ Hemoclips are the most commonly utilized hemostatic method; however, technical challenges arise, especially in ulcers with a rigid base, when attempting to capture spurting, oozing, or visible vessels.^[8]^ Despite technical and mechanical advancements in endoscopy, rebleeding rates reported in studies were 16% until 2020, however, data from the past 5 years indicate that this rate has decreased to less than 10%.^[9–13]^ The success of these methods, as in all endoscopic procedures, is closely linked to the skill and experience of endoscopists. The availability of multiple methods at the endoscopist’s discretion enhances treatment outcomes. Different techniques are especially beneficial for less experienced endoscopists, as access to additional treatment modalities improves success. From this perspective, hemosprays and the Ankaferd technique are noteworthy treatment alternatives, particularly for simplifying procedures for gastroenterologists and gastroenterology fellows with limited experience.^[14,15]^

The Ankaferd Blood Stopper (ABS) is a hemostatic agent prepared by combining 5 herbal ingredients. In Anatolia, the medicament formulation of the traditional treatment method for superficial bleeding, which has been transmitted among healers for centuries, is known and has been passed down through generations.^[16]^ It contains extracts of Thymus vulgaris, Glycyrrhiza glabra, Vitis vinifera, Alpinia officinarum, and Urtica dioica.^[17]^ Although the exact mechanism of action has not been fully elucidated, the primary hemostatic mechanism of ABS involves the formation of an encapsulated protein network that serves as a focal point for erythroid aggregation.^[18]^ The hemostatic effect of Ankaferd initiates in less than 1 second following application, as demonstrated under electron microscopy, inducing aggregation in all blood elements, notably erythroid elements.^[19]^ ABS, particularly over the last decade, has been increasingly employed as a hemostatic agent, especially in surgical dental procedures.^[20,21]^ Hemosprays, variously utilized worldwide in both primary and salvage interventions, have become a staple in the management of gastrointestinal system bleedings.^[22]^ In our study, we investigated the effect of ABS on procedural success in NVUGIB cases managed by gastroenterology fellows with 3 years or less of experience in upper endoscopy.

## 2. Method

Ethical approval for this study was obtained from the Ethics Committee of Ondokuz Mayis University Hospital (number 2021367). Between 2016 and 2021, a total of 653 patients who presented to our hospital’s emergency department (ED) with symptoms of acute upper gastrointestinal bleeding and whose Glasgow Blatchford Score was calculated as 6 or higher underwent urgent endoscopic procedures at the Gastroenterology Department Endoscopy Unit and were evaluated. A total of 202 patients who underwent endoscopic interventions performed by endoscopists with less than 3 years of experience were included. In our study, 9 endoscopists who received training in the Department of Gastroenterology at our University hospital performed all endoscopic bleeding interventions between 2015 and 2022. Emergency endoscopic interventions at our clinic can be performed after a 1-year training period. Gastroenterology fellows with 1 to 3 years of endoscopy experience performed all the endoscopies. The endoscopists used the same endoscopy equipment and auxiliary tools during the procedure; All endoscopic procedures were performed using Olympus CV-190 series endoscopes (Olympus, Tokyo, Japan). All of our hemoclips are SureClip™ hemoclips (Micro-Tech Nanjing Co., Ltd, Nanjing, Jiangsu, China), and the ERBE APC 2 Device was utilized for APC. All patients included in the study were intervened 12 to 24 hours after admission to the emergency department. Patients without active bleeding during endoscopy or with nonintervention-requiring findings were excluded from the study. European Society of Gastroenterology suggests employing combination therapy, involving epinephrine injection along with a secondary hemostasis modality (thermal or mechanical therapy), for patients with actively bleeding ulcers.^[5]^ In our study, dual hemostatic methods were employed for the majority of patients; endoscopists utilized combinations of adrenaline sclerotherapy, hemoclips, argon plasma coagulation, and ABS methods based on their clinical skills. An exception to this practice was observed in cases of bleeding due to malignancy, where endoscopists preferred either ABS or adrenaline sclerotherapy as a single method. Patients who received hemostasis treatment with ABS alone (ABS alone was used only in 14 out of 19 patients treated for gastric cancer bleeding. At least 2 methods were applied to all other patients with bleeding) or in combination were categorized as Group 1, while those who did not receive ABS and used single or combined methods were categorized as Group 2.

Demographic data, comorbidities, medication use, pre-procedure blood pressure, and pulse values of all included patients were recorded. Blatchford scores were calculated for all patients on admission to the ED. Hemogram parameters, bleeding time, blood urea nitrogen, and creatinine values were recorded for all patients before the procedure. During the procedure, the site of bleeding and interventions performed were documented. Transfusion requirements after patient admission were recorded, considering the initial hemoglobin levels, clinical conditions, and comorbidities. The hospitalization periods, both during admission and after discharge, were monitored for rebleeding, and any subsequent visits to our center or another facility due to bleeding were recorded. Rebleeding was characterized by the occurrence of fresh hematemesis and/or melena, coupled with the manifestation of shock or a decline in hemoglobin concentration exceeding 2 g/dL within a 24-hour period. Additionally, cases necessitating subsequent endoscopy, surgical intervention, or any interventional radiology procedure were also encompassed within the definition of rebleeding. Discharge decisions for patients included bleeding stabilization, additional comorbidities, bleeding site and severity, transfusion requirements, and clinical status. All patients were followed up for 14 days, rebleeding through the national patient tracking system and telephone contact.

### 2.1. Ankaferd blood stopper application

Ankaferd Blood Stopper (ABS), manufactured by AND Pharmaceuticals, is available in various sizes of pre-filled syringes and 200 mL bottles. Our clinic provides ABS in a 200 mL bottle form. It was drawn into standard 10 mL syringes and administered using standard 7F 180 cm sclerotherapy needles sent through the endoscope’s working channel. The sclerotherapy needle was held within the catheter, and ABS was sprayed onto the active bleeding or ulcer from a distance of 1 to 2 cm without injecting it into the tissue.

## 3. Results

The mean age of patients included in the study was 60.7 ± 17.4 years. Among the patients, 40.4% were female, and 59.6% were male. Table 1 provides the demographic data, medication use, additional risk factors, and presenting symptoms of the patients included in the study.

**Table 1:** Patients’ demographic information, comorbidities, bleeding sites, and types.

	Mean + SD	Min–max
Age	60.7 ± 17.4	62 (18–88)

	Frequency (n)	Percent (%)
Sex		
Woman	82	40.4
Man	120	59.6
Total	202	100
Smoking		
Yes	109	53.7
No	80	39.4
Ex-smoker	13	6.9
Total	202	100
CVD		
Yes	54	26.6
No	148	73.4
Total	202	100
Dementia		
Yes	4	2.0
No	198	98.0
Total	202	100
Liver cirrhosis		
Yes	14	7.4
No	188	92.6
Total	202	100
Kidney failure		
Yes	16	7.9
No	186	92.1
Total	202	100
Lung disease		
Yes	8	4.4
No	194	95.6
Total	202	100
NSAID		
Yes	76	37.4
No	126	62.6
Total	202	100
Oral anticoagulant		
Yes	52	26.1
No	150	73.9
Total	202	100
Bleeding site and type		
Bulbus ulcers	103	50.7
Gastric cancer	25	12.3
Mallory Weis Tear	17	8.4
GAVE	13	6.4
Stomach ulcer	38	19.2
Dielufoy lesion	6	3.0
Total	202	100

CVD = cardiovascular diseases, GAVE = gastric vascular antral ectasy, NSAID = non-steroidal anti-inflammatory drugs, SD = standard deviation.

The most frequently identified lesions during the endoscopic examination were bulbus ulcers, followed by gastric ulcers. Cardiovascular disease was the most common comorbidity. Approximately one-third of the patients used non-steroidal anti-inflammatory drugs. Melena was the most frequent presenting symptom, detected in 85.2% of patients during their ED admissions (Table 2).

**Table 2:** Clinical findings of patients at the time of admission.

	Frequency (n)	Percent (%)
Melena		
Yes	172	85.2
No	30	14.8
Total	202	100
Hematemesis		
Yes	44	21.7
No	158	78.3
Total	202	100
Hematochezia		
Yes	28	14.3
No	174	85.7
Total	202	100
Syncope		
Yes	46	23.2
No	156	76.8
Total	202	100
Blood pressure		
>100 mm Hg	126	62.6
<100 mm Hg	76	37.4
Total	202	100
Pulse		
>100	112	55.7
<100	90	44.3
Total	202	100
Hemoglobin		
>10 g/dL	114	56.7
<10 g/dL	88	43.3
Total	202	100

We divided the patients into 2 groups based on the method that used bleeding control during endoscopy. In Group 1, bleeding was treated with ABS (either alone or in combination with another method). In Group 2, bleeding was treated with methods other than ABS. Table 3 summarizes the general demographic characteristics of the groups, presenting symptoms, comorbidities, hospitalization duration, intensive care unit requirements, mortality, pre-procedure transfusion requirements, and pre-procedure laboratory investigations.

**Table 3:** Comparison of categorical variables by groups.

	Groups		Chi square	*P*
	Group 1	Group 2		
Sex				
Female	35 (36.5)	47 (44.3)	1.298	.255
Male	61 (63.5)	59 (55.7)		
Smoking				
Yes	41 (42.7)^a^	67 (63.2)^b^	31.090	**<.001**
No	55 (57.3)^a^	25 (23.6)^b^		
Ex smoker	0 (0)^a^	14 (13.2)^b^		
CVD				
Yes	26 (27.1)	28 (26.4)	0.011	.915
No	70 (72.9)	78 (73.6)		
HF				
Yes	3 (3.1)	5 (4.7)		.417^F^
No	93 (96.9)	101 (95.3)		
Dementia				
Yes	1 (1)	3 (2.8)		.349^F^
No	95 (99)	103 (97.2)		
Metastasis				
Yes	15 (15.6)	0 (0)	17.891	**<.001**
No	81 (84.4)	106 (100)		
Cirrhosis				
Yes	10 (10.4)	5 (4.7)	2.381	.123
No	86 (89.6)	101 (95.3)		
Kidney failure				
Yes	10 (10.4)	6 (5.7)	1.563	.211
No	86 (89.6)	100 (94.3)		
COPD				
Yes	4 (4.2)	5 (4.7)		.562^F^
No	92 (95.8)	101 (95.3)		
Syncope				
Yes	30 (31.2)	17 (16)	6.530	**.011**
No	66 (68.8)	89 (84)		
Hematemesis				
Yes	23 (24)	21 (19.8)	0.508	.476
No	73 (76)	85 (80.2)		
Hematochezia				
Yes	18 (18.8)	11 (10.4)	2.872	**.090**
No	78 (81.2)	95 (89.6)		
NSAID				
Yes	41 (42.7)	35 (33)	2.015	.156
No	55 (57.3)	71 (67)		
Oral anticoagulants				
Yes	30 (31.2)	23 (21.7)	2.375	.123
No	66 (68.8)	83 (78.3)		
Blood pressure				
>100 mm Hg	45 (46.9)	82 (77.4)	20.054	**<.001**
<100 mm Hg	51 (53.1)	24 (22.6)		
Pulse				
>100	30 (31.2)	83 (78.3)	45.250	**<.001**
<100	66 (68.8)	23 (21.7)		
Hemoglobin				
>10 g/dL	55 (57.3)	60 (56.6)	0.010	.921
<10 g/dL	41 (42.7)	46 (43.4)		
HTC				
>35	54 (56.3)	39 (36.8)	7.677	**.006**
<35	42 (43.8)	67 (63.2)		
INR				
<1.2	39 (40.6)	95 (89.6)	54.158	**<.001**
>1.2	57 (59.4)	11 (10.4)		
Blood transfusion				
Yes	79 (82.3)	63 (59.4)	12.605	**<.001**
No	17 (17.7)	43 (40.6)		
Bleeding source				
Bulbus ulcer	43 (44.8)	59 (55.7)	16.518	**.006**
Gastric cancer	19 (19.8)^a^	6 (5.7)^b^		
Mallory Weiss Tear	12 (12.5)^a^	5 (4.7)^b^		
GAVE	6 (6.3)	7 (6.6)		
Gastric ulcer	15 (15.6)	24 (22.6)		
Dieulafoy lesion	1 (1)	5 (4.7)		
Endoscopic hemostasis therapy				
ABS	14 (14.6)^a^	0 (0)^b^	202.000	**<.001**
APC + ABS	55 (57.3)^a^	0 (0)^b^		
Hemoclips + ABS	17 (17.7)^a^	0 (0)^b^		
AS + ABS	10 (10.4)^a^	0 (0)^b^		
Other	0 (0)^a^	106 (100)^b^		
Rebleeding				
Yes	3 (3.1)	8 (7.5)	1.913	.167
No	93 (96.9)	98 (92.5)		
ICU				
Yes	5 (5.2)	7 (6.6)	0.176	.675
No	91 (94.8)	99 (93.4)		
Mortality				
No	96 (100)	103 (97.2)		.143^F^
Yes	0 (0)	3 (2.8)		

ABS = Ankaferd blood stopper, APC = argon plasma coagulation, AS = adrenaline sclerotherapy, COPD = chronic obstructive pulmonary disease, CVD = cardiovascular disease, GAVE = gastric antral vascular ectasia, HF = heart failure, HTC = hematocrit, ICU = intensive care unit, INR = international normalized ratio, NSAID = nonsteroidal anti-inflammatory drugs.

Malignancy and metastasis were significantly higher in Group 1 than in Group 2. Furthermore, in the ABS group, hypotension, tachycardia, and syncope occurred significantly more frequently, suggesting a preference for ABS in more severe bleeding cases. Additionally, the higher pre-procedure transfusion requirement in the ABS group supports these findings. Three patients in group 2 experienced an adverse event during the one-month follow-up. The number of patients requiring intensive care due to bleeding was 12, with no statistical significance between the groups, although it was higher in group 2.

Of the 202 patients, 11 experienced recurrent bleeding within the first month, with 3 belonging to Group 1 and 8 to Group 2. While all patients treated with Ankaferd achieved acute hemostasis, 4 patients for whom other methods were preferred could not achieve hemostasis. Although not statistically significant, this finding was influential in the context of the small patient group. Ankaferd successfully achieved hemostasis in 4 patients for whom other methods could not control bleeding.

## 4. Discussion

Gastrointestinal bleeding is a significant public health issue that costs the healthcare system approximately 5 billion dollars annually.^[23]^ Although incidence rates vary significantly according to geographical location, previous studies consistently demonstrated a higher incidence among males and the elderly population.^[24,25]^ NVUGIB, which accounts for 25% to 67% of cases, is most frequently attributed to peptic ulcers, with melena being the most common presenting complaint among these patients.^[26]^ Similarly, in our study, approximately half (50.4%) of the patients who underwent bleeding interventions had bleeding originating from duodenal ulcers, and most of our patients presented with melena (85.2%). Given the continued prevalence of gastrointestinal bleeding as a medical condition, proper management of this emergency will significantly affect mortality and morbidity.^[23]^ Endoscopic treatment is the cornerstone of treatment for gastrointestinal bleeding. The diversity of available methods in the management of treatment, selection of an appropriate method for a suitable patient, endoscopist’s experience, and ease of the chosen method are significant factors influencing treatment success.^[27,28]^ The use of endoscopic treatment methods for gastrointestinal bleeding is increasing daily. The ease of application of these methods particularly affects treatment success, especially for inexperienced gastroenterologists. Our study evaluated the impact of the Ankaferd Blood Stopper, used during endoscopic interventions for NVUGIB, on the cessation of gastrointestinal bleeding among gastroenterologists with less than 3 years of endoscopy experience. Hemosprays are commonly used as rescue or salvage therapy for gastrointestinal bleeding. Our study predominantly employed ABS for salvage and bleeding stabilization purposes. A recent meta-analysis, comprising 20 studies involving 1280 patients, reported a clinical success rate of 91% (95% CI 88–94, *I*^2^ = 47.72%) for hemospray compared to 87% (95% CI 75–94, *I*^2^ = 0.00%) for other hemostatic measures.^[29]^ Our study achieved acute hemostasis in all patients treated with ABS, suggesting that ABS may have comparable or even higher effectiveness than other hemosprays. The early rebleeding rates for hemospray ranged from 8% to 31% in this meta-analysis, whereas in our study, this rate was much lower for Ankaferd (3.1%). Comparative studies between ABS and other hemosprays would be beneficial to better assess this difference.^[29]^ In our study, early rebleeding and the need for repeat endoscopy were lower in patients treated with ABS, indicating its effectiveness compared with other methods. Among the 3 patients who experienced rebleeding after ankaferd treatment, two had interventions for gastric antral vascular ectasia, suggesting that the efficacy of ABS may be limited in cases of vascular ectasias. In our study, a statistically significant difference in rebleeding rates could not be identified; however, it is noteworthy that the numerical incidence of rebleeding was lower in the group where ABS was used. On the other hand, it is worth mentioning that endoscopists may have preferred ABS as a salvage method in many cases since they did not use it randomly. Achieving a 100% success rate for acute hemostasis is a pivotal advantage. Notably, the prompt attainment of acute hemostasis facilitates timely referral to larger medical centers and is a substantial means of averting protracted procedural complications. Intervention for upper gastrointestinal bleeding requires knowledge and experience. Unsuccessful attempts prolong procedural duration, particularly decreasing patient compliance in non-anesthetized individuals and elevating anesthesia-related complications in anesthetized patients, thereby increasing risks such as aspiration adversely.^[30]^ Multiple treatment options, especially for young endoscopists, enhance the likelihood of treatment success.^[31]^ ABS’s non-contact application provides extensive hemostasis in the applied area and represents a significant treatment method owing to its rapid hemostatic effect post-application.^[32,33]^ Tumoral sources contribute to approximately 5% of gastrointestinal bleeding cases. Gastrointestinal tumor-related bleeding often occurs over a broad area and presents as oozing, which contributes to higher rebleeding rates. Gastrointestinal tumor-related bleeding is typically challenging to control using conventional endoscopic hemostasis methods because of their tendency to spread over a wide area, diffuse bleeding from multiple sites, and the advanced fragility of their surface.^[34–36]^ A study that assessed Hemospray in gastrointestinal tumor bleeding showed that the rate of rebleeding was 3 times higher in the group that did not use Hemospray than in the group that used it.^[37]^ Our study successfully achieved hemostasis with ABS in all 19 patients with tumor bleeding, and none of them experienced rebleeding during the 14-day follow-up period. Future studies comparing classical hemostatic methods, other hemostatic sprays, and ABS in advanced-stage gastrointestinal tumor bleeding may provide more detailed information.

Our study had some limitations; firstly, it was conducted at a single center and the number of cases was limited, hence our results cannot be generalized to the population. Since our endoscopy unit also serves as a training clinic, all procedures were performed by endoscopists with 1 to 3 years of clinical experience, which may affect the success rates compared to interventions performed by more experienced endoscopists. In our study, all cases of peptic ulcer bleeding were intervened with at least 2 methods. Patients who received only ABS or only AS were all due to bleeding caused by gastric cancer, so this group was evaluated separately but not excluded from the overall statistics. As seen in Table 3, there was a significant difference in terms of disease history in the group where ABS was preferred, and we believe that this selection bias may have occurred because endoscopists considered ABS as a salvage therapy and preferred it in more difficult cases and cases where bleeding control was more challenging.

## 5. Conclusion

ABS, applied by gastroenterologists with less than 3 years of endoscopy experience, either alone or in combination with other methods, demonstrates notable procedural success. Our study found favorable outcomes, achieving hemostasis during the initial procedure and reducing rebleeding rates, particularly in gastrointestinal tumors. Although limited by its single-center design and case number, future prospective studies involving multiple centers and larger cohorts can offer more meaningful data.

## Author contributions

**Conceptualization:** Berk Bas.

**Data curation:** Berk Bas.

**Formal analysis:** Berk Bas.

**Funding acquisition:** Berk Bas.

**Investigation:** Berk Bas.

**Resources:** Berk Bas.

**Software:** Berk Bas.

**Supervision:** Muge Ustaoglu.

**Visualization:** Berk Bas, Omer Kucukdemirci, Muge Ustaoglu.

**Writing – original draft:** Berk Bas.

**Writing – review & editing:** Berk Bas, Omer Kucukdemirci.
